# Polo-like kinase 1 inhibition diminishes acquired resistance to epidermal growth factor receptor inhibition in non-small cell lung cancer with *T790M* mutations

**DOI:** 10.18632/oncotarget.10332

**Published:** 2016-06-30

**Authors:** Yuehong Wang, Ratnakar Singh, Liguang Wang, Monique Nilsson, Ruchitha Goonatilake, Pan Tong, Lerong Li, Uma Giri, Pamela Villalobos, Barbara Mino, Jaime Rodriguez-Canales, Ignacio Wistuba, Jing Wang, John V. Heymach, Faye M. Johnson

**Affiliations:** ^1^ Department of Respiratory Medicine, The First Affiliated Hospital, College of Medicine, Zhejiang University, Hangzhou, China; ^2^ Department of Thoracic/Head and Neck Medical Oncology, The University of Texas MD Anderson Cancer Center, Houston, Texas, USA; ^3^ Institute of Oncology, Provincial Hospital Affiliated to Shandong University, Shandong University, Jinan, China; ^4^ Department of Bioinformatics and Computational Biology, The University of Texas MD Anderson Cancer Center, Houston, Texas, USA; ^5^ Department of Translational Molecular Pathology, The University of Texas MD Anderson Cancer Center, Houston, Texas, USA; ^6^ The University of Texas Graduate School of Biomedical Sciences, Houston, Texas, USA

**Keywords:** polo-like kinase, epidermal growth factor receptor, drug resistance, non-small cell lung cancer, epithelial–mesenchymal transition

## Abstract

Epidermal growth factor receptor (EGFR) tyrosine kinase inhibitors (TKIs) are effective against non-small cell lung cancer (NSCLC) with activating *EGFR* mutations, but resistance is inevitable. Mechanisms of acquired resistance include *T790M* mutations and epithelial–mesenchymal transition (EMT). One potential strategy for overcoming this resistance is the inhibition of polo-like kinase 1 (PLK1) based on our previous studies showing that mesenchymal NSCLC cell lines are more sensitive to PLK1 inhibition than epithelial cell lines. To determine the extent to which PLK1 inhibition overcomes EGFR TKI resistance we measured the effects of the PLK1 inhibitor volasertib alone and in combination with the EGFR inhibitor erlotinib *in vitro* and *in vivo* in *EGFR* mutant NSCLC cell lines with acquired resistance to erlotinib. Two erlotinib-resistant cell lines that underwent EMT had higher sensitivity to volasertib, which caused G2/M arrest and apoptosis, than their parental cells. In all NSCLC cell lines with *T790M* mutations, volasertib markedly reduced erlotinib resistance. All erlotinib-resistant NSCLC cell lines with *T790M* mutations had higher sensitivity to erlotinib plus volasertib than to erlotinib alone, and the combination treatment caused G2/M arrest and apoptosis. Compared with either agent alone, the combination treatment also caused significantly more DNA damage and greater reductions in tumor size. Our results suggest that PLK1 inhibition is clinically effective against NSCLC that becomes resistant to EGFR inhibition through EMT or the acquisition of a *T790M* mutation. These results uncover new functions of PLK1 inhibition in the treatment of NSCLC with acquired resistance to EGFR TKIs.

## INTRODUCTION

Epidermal growth factor receptor (EGFR) tyrosine kinase inhibitors (TKIs) are effective therapy against advanced non-small cell lung cancer (NSCLC) with activating *EGFR* mutations [1–3]. Compared with patients with this disease who receive standard chemotherapy, those who receive treatment with EGFR TKIs have longer progression-free survival and better quality of life [1, 2]. However, the disease inevitably acquires resistance to EGFR TKIs. Mechanisms of this resistance include the development of a second-site *EGFR* resistance mutation (*T790M*); the activation of parallel signaling pathways, including cMET, HER2, FGFR, Mer and AXL; up regulation of SCRN1; epithelial–mesenchymal transition (EMT); and small cell transformation [4–11]. Drugs that inhibit glutaminase C, JAK, Mer and Src are effective in some EGFR TKI resistance models [8–10, 12]. In addition, resistance to EGFR inhibition can result from an innate mechanism whereby the short-term inhibition of downstream AKT leads to decreased Ets1 expression and subsequently decreased levels of DUSP6, the negative regulator of ERK1/2 [13]. Patients who have NSCLC with acquired resistance to EGFR TKIs have limited therapeutic options. Although AZD9291 has efficacy against NSCLC with *T790M* mutations, effective strategies for overcoming other resistance mechanisms are lacking [4, 14]. Therefore, there is an urgent need for developing new effective treatments to overcome or delay acquired resistance to EGFR TKIs.

One potential strategy to overcome acquired resistance to EGFR TKIs is the inhibition of polo-like kinase 1 (PLK1). PLK1, which is overexpressed in various malignancies, including NSCLC, regulates many cell cycle events, including mitotic entry, centrosome maturation, kinetochore assembly, and bipolar spindle formation. It also modulates DNA damage responses, including the recovery of DNA damage checkpoints, and contributes to oncogenesis by inducing chromosome instability. Inhibiting PLK1 in NSCLC with acquired EGFR TKI resistance has been investigated previously. Crystal et al. subjected NSCLC cells with acquired EGFR TKI resistance to genetic and pharmacologic screens and identified diverse drug sensitivities in the resulting models. They found that although most erlotinib-resistant (ER) cell lines were not sensitive to the 76 agents tested, the PLK1 inhibitor BI2536 was effective against five ER NSCLC cell lines and two patient-derived cell lines [15]. However, the authors did not investigate the mechanism underlying the PLK1 inhibitor's action. Our own studies revealed that mesenchymal NSCLC cell lines are more sensitive to PLK1 inhibition than epithelial cell lines are *in vitro* and *in vivo*, indicating that PLK1 inhibition may be effective against NSCLC with acquired EGFR-TKI resistance that had undergone EMT. We hypothesize that PLK1 inhibition may be effective in NSCLC with acquired EGFR-TKI resistance that had undergone EMT based on our own previous studies showed that mesenchymal NSCLC cell lines are more sensitive to PLK1 inhibition than epithelial cell lines are *in vitro* and *in vivo* [16]. Other studies have shown that both NSCLC cell lines and patient tumors undergo EMT when they acquire resistance to EGFR TKIs [15, 17–21]. For example, HCC827 cells resistant to the EGFR TKI gefitinib produced transforming growth factor beta 1 (TGF-β1), and when parental HCC827 cells were exposed to TGF-β1, they underwent EMT and became resistant to gefitinib; however, the suppression of EMT did not prevent this acquired resistance [17]. In addition, PLK1 inhibition has been shown to significantly augment the anti-tumor effect of EGFR inhibitors in EGFR inhibition–resistant glioblastoma cell lines harboring EGFRvIII mutations [22].

PLK1 regulates many cell cycle events, including mitotic entry, centrosome maturation, kinetochore assembly, and bipolar spindle formation [23]. In addition to governing mitotic progression, PLK1 also modulates DNA damage responses, including the recovery of DNA damage checkpoints. PLK1 is overexpressed in various malignancies, including NSCLC, melanoma, colorectal cancer, and prostate cancer, and contributes to oncogenesis by inducing chromosome instability [24, 25]. PLK1 levels in NSCLC are correlated inversely with survival [26]. In cancer cells, the knock down [27] or inhibition of PLK1 results in a variety of biological effects, including G2/M accumulation, spindle defects, chromosomal alignment defects, mitotic slippage, apoptosis, senescence, and defective centrosome maturation or separation [28–31]. Among the PLK1 inhibitors in clinical trials, volasertib (BI6727) has received breakthrough status for the treatment of acute myeloid leukemia from the U.S. Food and Drug Administration and is being studied in different malignancies including NSCLC [32, 33].

One limitation of using single-agent PLK1 inhibition to treat ER NSCLC is that there are multiple, diverse mechanisms of acquired resistance to EGFR inhibitors. In addition, single tumors may have multiple mechanisms of resistance simultaneously due to heterogeneity [17]. Finally, as Crystal et al. found, single agents were not effective in the vast majority of ER NSCLC models they developed [15]. In the present study, therefore, we tested the combination of the EGFR inhibitor erlotinib and the PLK1 inhibitor volasertib in ER NSCLC models to determine the extent to which PLK1 inhibition overcomes EGFR TKI resistance.

On the basis of the above findings, we hypothesize that PLK1 inhibition is effective against NSCLC with acquired EGFR-TKI resistance that undergone EMT and may reverse EGFR-TKI resistance. In the current study, we measured the effects of the PLK1 inhibitor volasertib alone and in combination with the EGFR inhibitor erlotinib in a panel of ER NSCLC cells lines *in vitro* and *in vivo*. We also investigated the mechanism underlying the observed synergistic effects of the drugs.

## RESULTS

### Erlotinib resistant NSCLC cell lines have higher PLK1 expression and greater sensitivity to PLK1 inhibition

**Figure 1:**
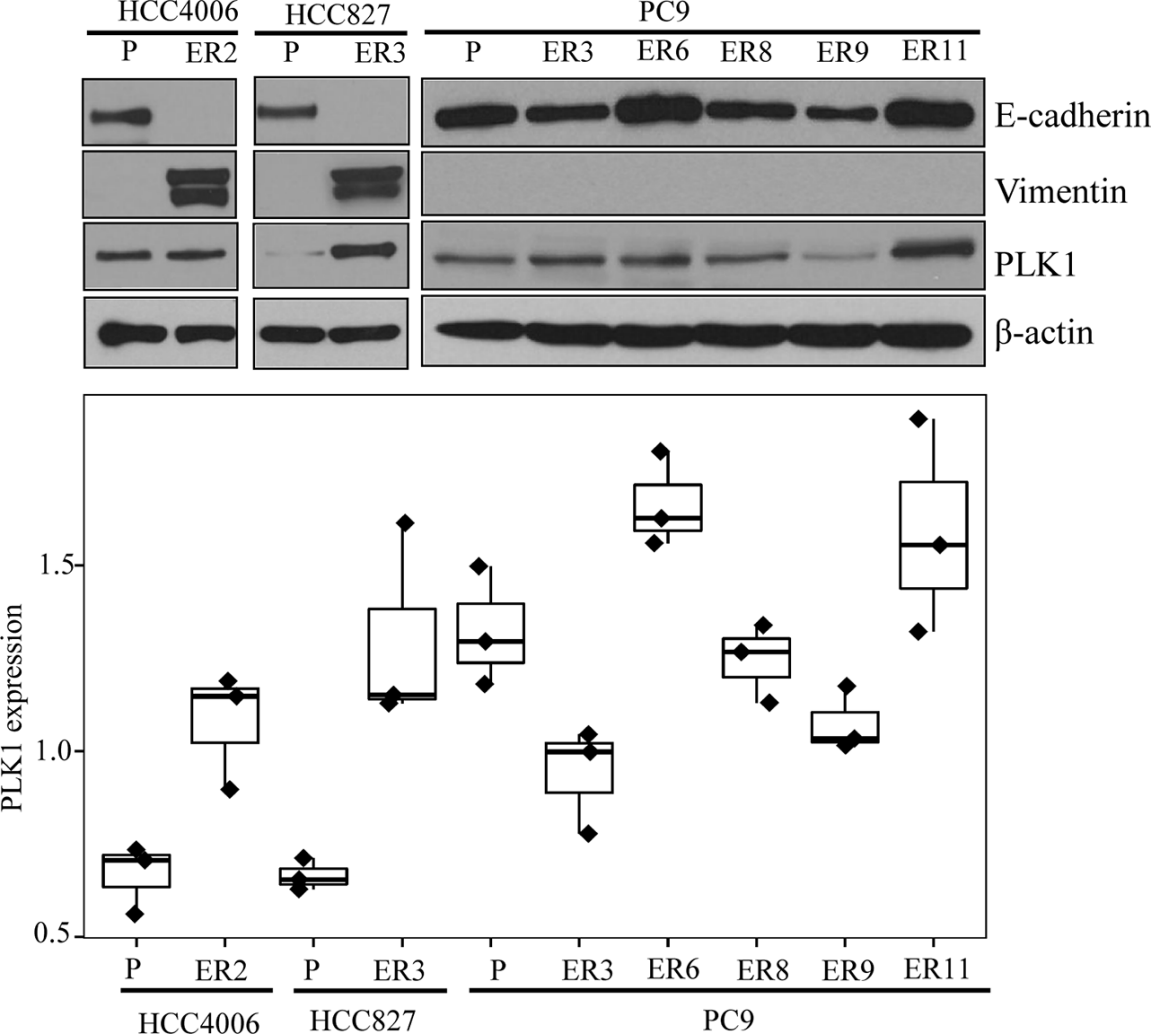
Characteristics of parental and ER NSCLC cell lines Parental and ER NSCLC cell lines were assessed for protein expression with Western blotting and for viability with the CellTiter-Glo assay. E-cadherin, vimentin, and PLK1 protein expression from the Western blot analysis (**A**) were quantitated using the Image J software program (**B**).

Three cell lines with endogenous *EGFR* mutations (PC9, HCC4006, and HCC827) were previously developed (MN, JVH) by exposure to serial dilutions of erlotinib *in vitro* to establish 7 NSCLC cell lines with acquired erlotinib resistance (ER). Five ER clones had secondary *T790M EGFR* mutations and two did not. Western blotting for E-cadherin and vimentin expression was consistent with EMT in those 2 ER lines lacking a *T790M EGFR* mutation (Figure 1). A CellTiter-Glo assay assessing the cells' sensitivity to erlotinib confirmed maintenance of the ER phenotype (Table 1).

**Table 1:** Drug sensitivity of parental cell lines and cell lines with acquired erlotinib resistance

Cell line	Volasertib			Erlotinib		
	IC_50_(μM)	IC_70_(μM)	AUC	IC_50_(μM)	IC_70_(μM)	AUC
**PC9**	0.03	0.05	0.30	0.50	0.50	0.02
**PC9-ER3**	0.06	0.10	0.54	2.41	2.93	0.71
**PC9-ER6**	0.06	0.10	0.57	3.07	3.76	0.70
**PC9-ER8**	0.05	0.08	0.46	2.28	4.00	0.59
**PC9-ER9**	0.05	0.16	0.57	3.46	3.99	0.83
**PC9-ER11**	0.05	0.16	0.51	2.53	4.00	0.63
**HCC4006**	0.16	0.16	0.61	0.50	3.61	0.34
**HCC4006-ER2**	0.03	0.04	0.31	4.00	4.00	0.69
**HCC827**	0.02	0.16	0.45	0.50	0.50	0.08
**HCC827-ER3**	0.03	0.03	0.22	4.00	4.00	0.95

Abbreviations: IC_50_: half maximal inhibitory concentration; IC_70_: 70 percent inhibitory concentration; AUC: Area Under Curve

**Figure 2:**
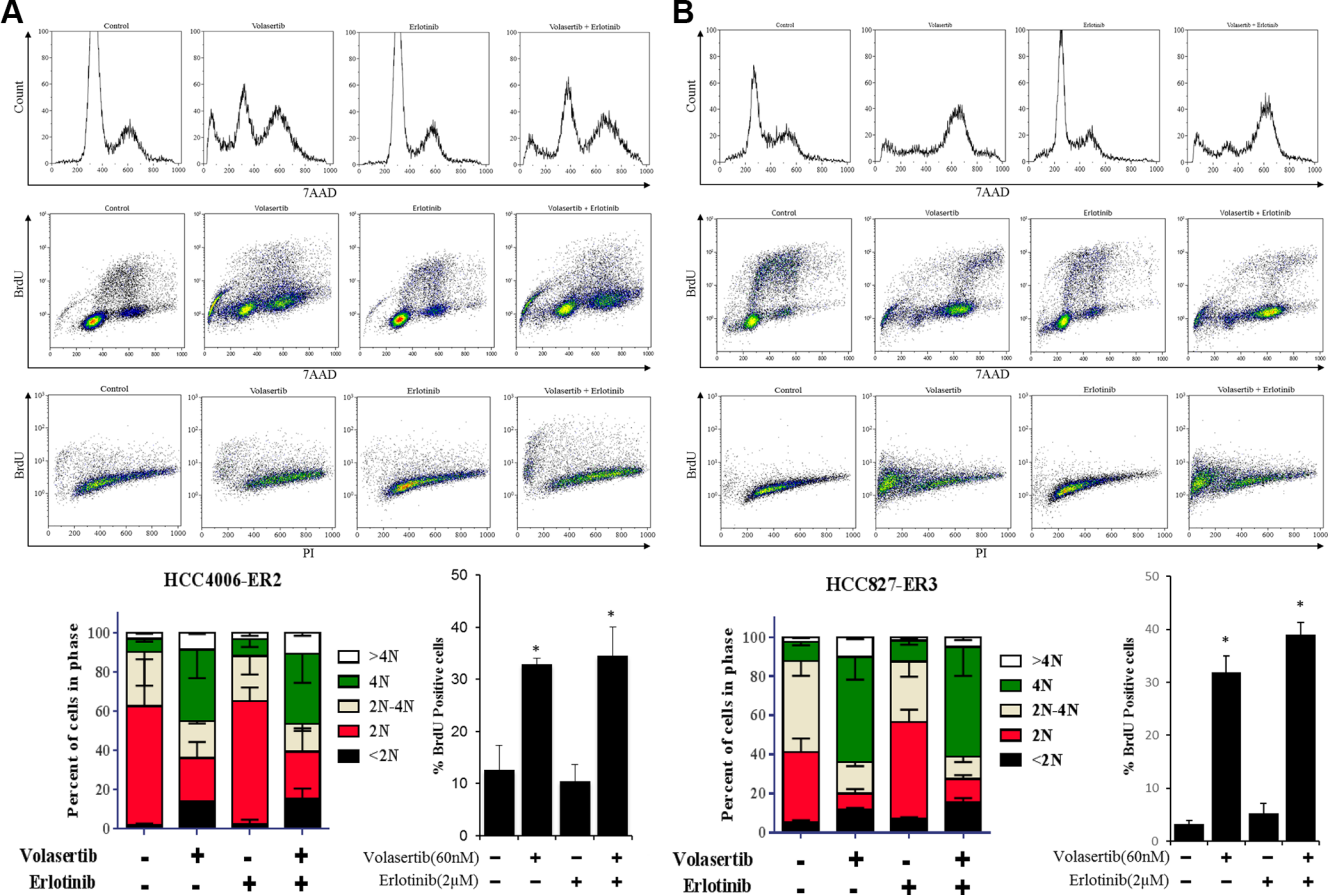
PLK1 inhibition is sufficient to induce cell cycle arrest and apoptosis in NSCLC cell lines that have undergone EMT HCC4006-ER2 (**A**) and HCC827-ER3 (**B**) cell lines were treated with 60 nM volasertib and/or 2 μM erlotinib or with vehicle controls and the cell cycle was analyzed with a BrdU FITC flow cytometry kit with 7AAD after 48 h. Apoptosis was assessed with an APO-BrdU TUNEL assay. *p < 0.05 compared with vehicle control after 72 hours.

Our prior research demonstrated that mesenchymal NSCLC cell lines are more sensitive to PLK1 inhibitors than epithelial NSCLC cell lines are. In the present study, therefore, we used the CellTiter-Glo assay to assess ER clones' sensitivity to the PLK1 inhibitor volasertib (Table 1, Supplementary Figure 1). We calculated the IC_50_, IC_70_, and area under the dose response curve (AUC) values of volasertib because the dose response curves often plateau at or near the IC50 value and because we previously found that the IC70 and AUC values are better discriminators of sensitivity [16]. The two ER lines that underwent EMT were more sensitive to volasertib after undergoing EMT. We tested the effect of 60 nM volasertib in two of these lines (HCC4006-ER2 and HCC827-ER3) and found that the agent caused marked G2/M arrest and apoptosis (Figure 2), which supports our hypothesis that EMT enhances ER NSCLC cells' sensitivity to PLK1 inhibitors.

Compared with parental cell lines, most ER cell lines with diverse mechanisms of resistance had significantly greater PLK1 protein expression (Figure 1). Increased volasertib sensitivity was not correlated with increased PLK1 expression (*p* = 0.81).

### PLK1 and EGFR inhibitors are synergistic and cause apoptosis in NSCLC cells bearing *T790M EGFR* mutations

**Figure 3:**
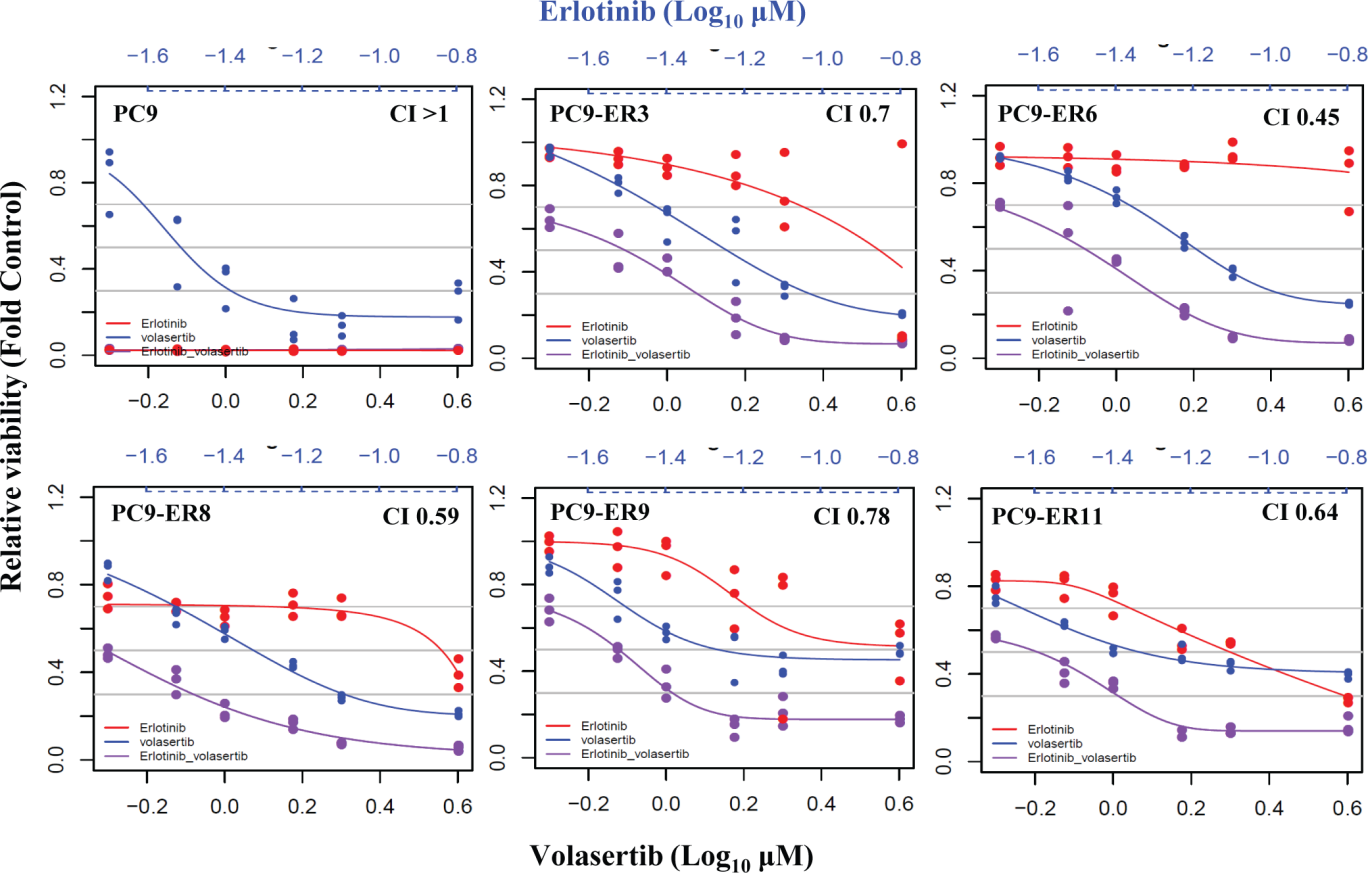
PLK1 inhibition plus EGFR inhibition is synergistic in ER PC9 cell lines harboring *T790M EGFR* mutations The viability of ER PC9 cell lines treated with volasertib and/or erlotinib for 72 h was assessed with the CellTiter-Glo assay. The CI of the two drugs was calculated using the Calcusyn software program. CI depicts synergism (CI < 1), additive effect (CI = 1), and antagonism (CI > 1).

As single agents are not effective in the vast majority of ER NSCLC models [15], we investigated the effect of combined PLK1 and EGFR inhibition on ER cell lines. The combination of volasertib and erlotinib was synergistic in all ER clones with *T790M* mutations (i.e., PC9-ER clones; Figure 3). In contrast, the combination was largely antagonistic in ER cell lines that had undergone EMT, although volasertib alone was effective in these cell lines, as noted above (Supplementary Figure 2).

**Figure 4:**
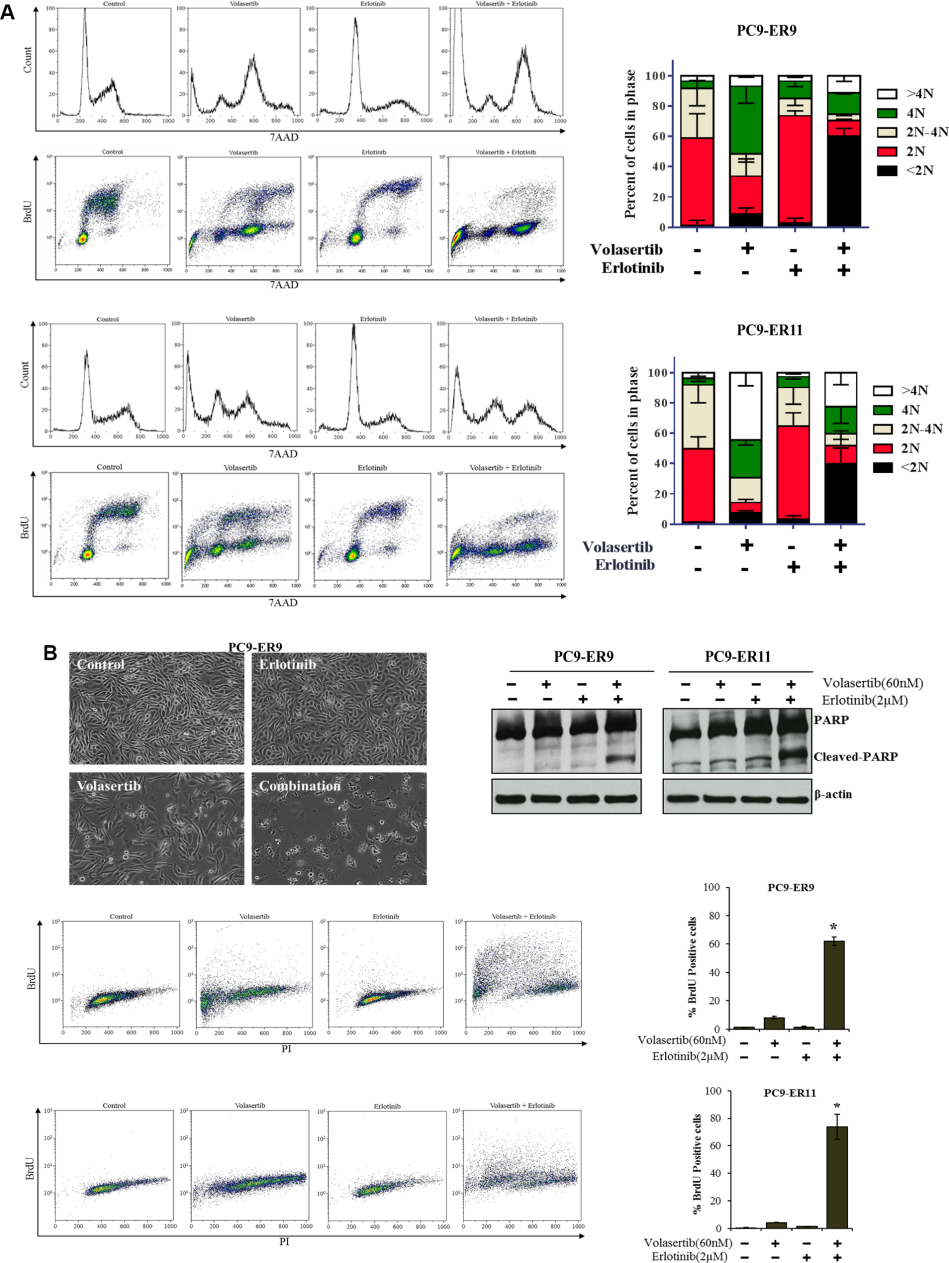
PLK1 inhibition plus EGFR inhibition induces cell cycle arrest and apoptosis in ER PC9 cell lines harboring *T790M EGFR* mutations PC9-ER9 and PC9-ER11 cell lines were treated with 60 nM volasertib and/or 2 μM erlotinib or with vehicle controls for 48 h (**A**) or 72 h (B). (A) The cell cycle was analyzed with a BrdU FITC flow cytometry kit with 7AAD. (**B**) Apoptosis was analyzed by assessing typical morphological changes in PC9-ER9 cells (upper left); performing a APO-BrdU TUNEL assay (lower panels); and performing Western blotting for cleaved PARP levels (upper right). *p < 0.05 compared with single-agent volasertib or erlotinib.

We assessed the drug combination's effect on the cell cycle and apoptosis in two cell lines against which the agents acted synergistically (PC9-ER9 and PC9-ER11) and in two cell lines against which the agents did not act synergistically (HCC4006-ER2 and HCC827-ER3) using relevant drug concentrations of 60 nM volasertib and 2 μM erlotinib [33, 34]. Despite erlotinib resistance, erlotinib still induced G1 arrest and a reduction in proliferation (S phase). As expected, volasertib by itself induced G2/M arrest and increased polyploidy (> 4N). However, ER PC9 cell lines treated with volasertib plus erlotinib had a pronounced sub-G0 fraction as well as polyploid cells (Figure 4A). The cell cycle patterns of the HCC4006-ER2 and HCC827-ER3 cell lines treated with the combination were similar to those of the HCC4006-ER2 and HCC827-ER3 cell lines treated with volasertib alone (Figure 2).

Consistent with the increase of the sub-G0 fraction, substantial apoptosis, as measured by assessing PARP cleavage and performing a TUNEL assay, occurred in the ER PC9 cell lines treated with the combination of volasertib and erlotinib. Single agents did not cause significant apoptosis (Figure 4B), which was consistent with the results of the CellTiter-Glo assay. Apoptosis in the HCC4006-ER2 and HCC827-ER3 clones was significantly increased after treatment with volasertib alone but was not enhanced with erlotinib treatment (Figure 2).

### PLK1 inhibition plus EGFR inhibition enhances DNA damage in ER NSCLC cells bearing *T790M EGFR* mutations

**Figure 5:**
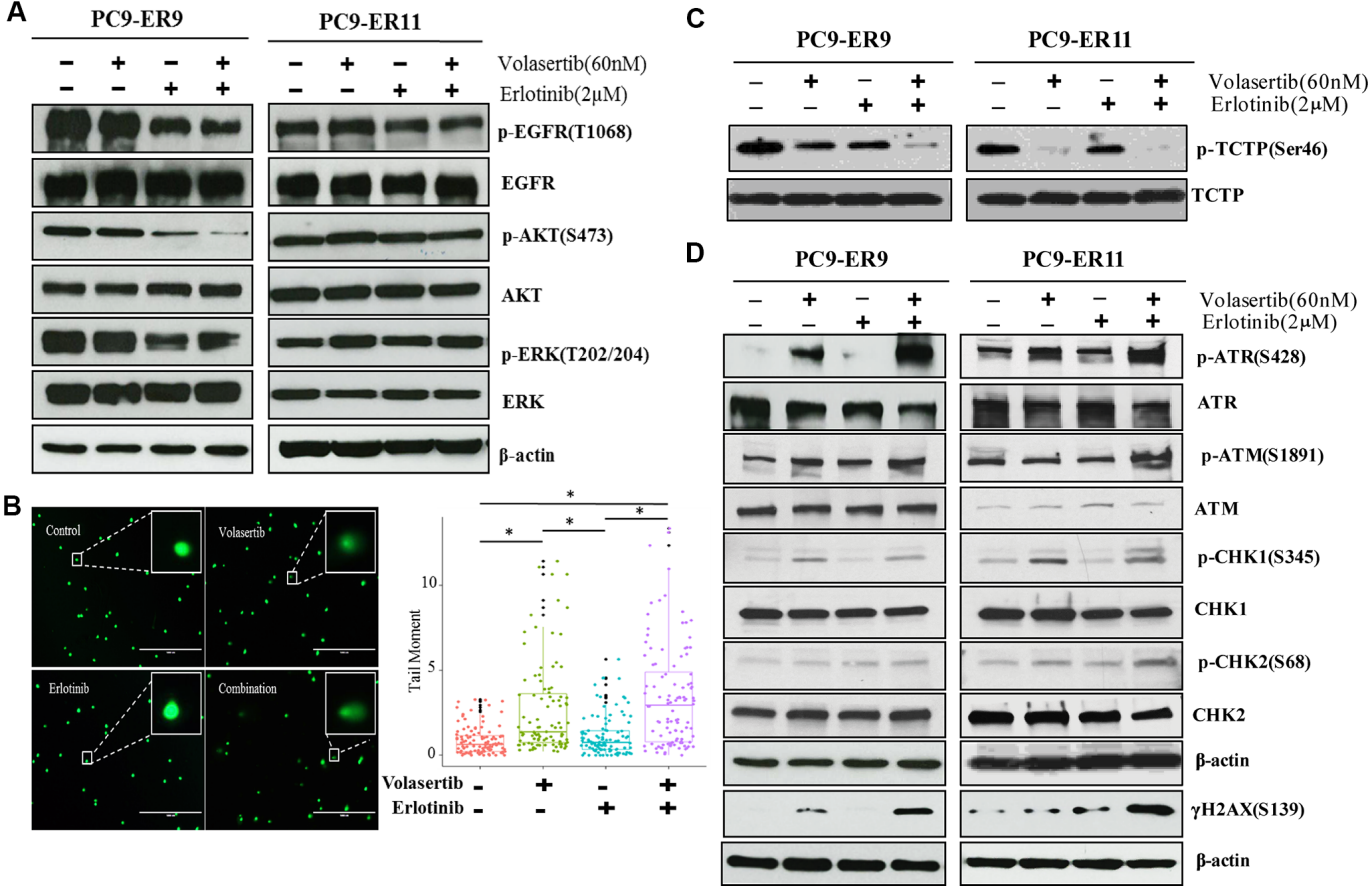
PLK1 inhibition plus EGFR inhibition induces DNA damage but does not substantially affect canonical EGFR downstream pathways in ER PC9 cell lines harboring *T790M EGFR* mutations PC9-ER9 and PC9-ER11 cell lines were treated with 60 nM volasertib and/or 2 μM erlotinib or with vehicle controls for 2 h (**A**) or 48 h (**B–D**). (A, C, D) Cells were lysed and subjected to Western blotting with the indicated antibodies. (B) Representative fluorescence microscopy images of a comet assay (left panel); quantification comet tail moment in individual cells (right panel). *p < 0.05.

Acquired resistance to EGFR TKIs often results in the reactivation of signaling pathways downstream of EGFR, including the PI3K/AKT and Ras/ERK pathways [4, 5]. To determine the extent to which the addition of PLK1 inhibition enhances the effects of EGFR inhibition on canonical downstream signaling pathways, we treated PC9-ER9 and PC9-ER11 cells with erlotinib and/or volasertib and used Western blotting to assess the expression of key signaling proteins (Figure 5A). Volasertib alone did not affect the activation of EGFR, AKT, or ERK. Despite the cells' *T790M* mutations, erlotinib inhibited EGFR signaling modestly. The addition of PLK1 inhibition did not enhance the inhibitory effect of EGFR inhibition on ERK in either cell line but did enhance this effect on AKT in PC9-ER9 cells. This result demonstrates that the dramatic synergy of volasertib and erlotinib did not result from the bypassing of EGFR to enhance the inhibition of PI3K/AKT or Ras/ERK.

PLK1 regulates the DNA damage response checkpoint [35, 36], and PLK1 inhibition can cause DNA damage [28, 29, 31]. To determine the extent to which PLK1 inhibition induces DNA damage in ER NSCLC cells, we incubated the cells with volasertib or erlotinib for 48 h and then subjected them to a comet assay which measures both single-strand and double-strand DNA breaks (Figure 5B). Erlotinib alone did not induce increased DNA damage, but volasertib alone did. The combination treatment also enhanced DNA damage. To determine whether the DNA damage response was engaged, we used Western blotting to measure γ-H2AX, CHK1/ATR, and CHK2/ATM expression (Figure 5D). The expression of γ-H2AX, p-ATR, and p-CHK1 was modestly increased after volasertib treatment; however, the co-administration of volasertib and erlotinib enhanced the phosphorylation of these proteins.

### Inhibition of both PLK1 and EGFR is more effective than inhibition of either target alone in NSCLC xenograft models

**Figure 6:**
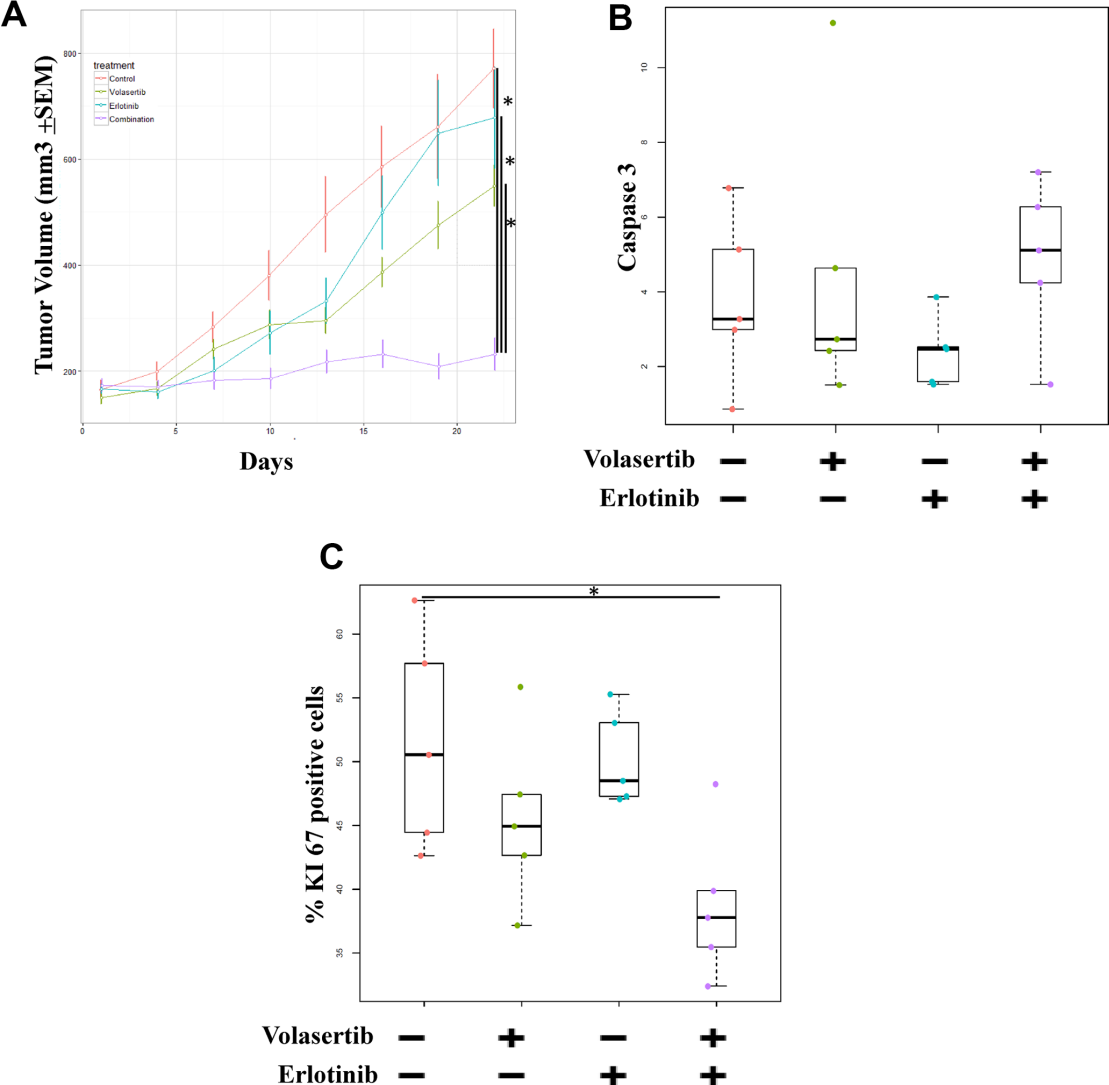
Inhibition of both PLK1 and EGFR is more effective than inhibition of either target alone in ER NSCLC xenograft models. Mice bearing PC9-ER9 xenograft tumors with volumes of 150 mm^3^ were treated with volasertib and/or erlotinib or with vehicle controls (**A**) Tumor volume was measured twice weekly and significantly decreased after combination treatment. After 3 weeks of treatment, the mice were humanely killed. Resected tumors were subjected to immunohistochemical analysis for Ki67 (**C**) and caspase 3 (**B**) protein expression, which was scored and quantitated.

We then assessed the activity of PLK1 and EGFR inhibition in a PC9-ER9 xenograft model. Compared with the control treatment, treatment with volasertib or erlotinib alone did not inhibit tumor growth (*p >* 0.05). However, compared with treatment with either agent alone, the combination treatment significantly reduced tumor size (*p* < 0.01, Figure 6A). To determine the inhibitory effect of the treatments on cell proliferation and apoptosis in the xenograft models, we measured the expression of Ki67 and caspase-3 protein by immunohistochemistry. Caspase 3 staining for the combination treatment group was higher than that for either single-agent treatment group, but this difference was not significant (Figure 6B). Consistent with the different treatments' effects on the cell cycle, Ki-67 staining for the single-agent treatment groups did not differ significantly (*p* > 0.05), but Ki-67 staining for the combination treatment group was significantly lower than that for either single-agent treatment group (*p* = 0.03) (Figure 6C).

## DISCUSSION

In this study, we found that ER NSCLC cell lines were more sensitive to PLK1 inhibition after undergoing EMT. ER NSCLC cell lines that acquired *T790M EGFR* mutations were more resistant to single-agent PLK1 inhibitors than the parental cell lines were, but the addition of PLK1 inhibition reversed erlotinib resistance. The combination treatment led to DNA damage in ER NSCLC cell lines harboring *T790M EGFR* mutations marked apoptosis in the ER cell lines with *T790M* mutations, and decreased tumor growth in mice.

To our knowledge, only one other study has shown that PLK1 inhibition is effective in ER NSCLC models [15]. That study's results are consistent with those of the present study, in that a minority of the models were sensitive to PLK1 inhibition. In the current study, all ER NSCLC cell lines that became more sensitive to PLK1 inhibition had undergone EMT, an established mechanism of acquired EGFR TKI resistance [18, 20]. Currently, there is no effective strategy for overcoming EGFR TKI resistance that is gained via EMT. One potential strategy is to inhibit the receptor tyrosine kinase AXL [21], but this approach is not universally effective [37]. Our previous study showed that mesenchymal NSCLC cell lines were more sensitive to three PLK1 inhibitors (BI2536, volasertib, and GSK461364) than epithelial cell lines were both *in vitro* and *in vivo*. In addition, the manipulation of EMT status using TGF-β or ectopic expression of miR200 or ZEB1 altered cells' sensitivity to PLK1 [16].

Surprisingly, volasertib and erlotinib had strikingly synergy against NSCLC cells with the *T790M* mutation, despite the fact that these cell lines were more resistant to the single agents than the parental line was. The *T790M* mutation is the dominant mechanism of acquired EGFR TKI resistance, accounting for more than half of the cases of NSCLC resistant to the drugs. The *T790M* mutation in EGFR decreases the affinity of EGFR TKIs to the ATP-binding pocket of the catalytic region and reactivates the EGFR signaling pathway [37]. Several mutant-specific EGFR TKIs, such as AZD9291, CO-1686, ASP8273, and EGF816, are effective against *T790M*-mutant NSCLC. AZD9291 was recently approved by the U.S. Food and Drug Administration for the treatment of NSCLC patients with acquired *T790M* mutations [38, 39]. Our finding that PLK1 inhibition combined with erlotinib could overcome the EGFR-TKI resistance with *T790M* mutation will provide new treatment strategy for the acquired EGFR-TKI resistance in NSCLC.

In the present study, volasertib had no inhibitory effect on the canonical EGFR signaling pathways (i.e., MEK/ERK or AKT). Erlotinib alone had partial inhibitory effects on EGFR, ERK, and AKT phosphorylation, which is consistent with prior studies showing that the *T790M* mutation typically occurs in a minor population of EGFR TKI-resistant cells and that EGFR TKI–resistant cells retain drug-sensitive alleles. However, erlotinib did not completely inhibit downstream AKT and ERK, which is consistent with previous studies' findings that NSCLC with the *T790M* mutation is resistant to EGFR TKIs [40, 41]. The combination of PLK1 and EGFR inhibition did not consistently inhibit ERK or AKT more than EGFR inhibition alone did, suggesting that the PLK1 pathway does not simply serve as “bypass” of EGFR-dependent pathways to facilitate resistance to EGFR inhibition.

The present study demonstrated that PLK1 inhibition combined with erlotinib significantly enhanced DNA damage and apoptosis. PLK1 has a pivotal role in maintaining mitotic entry and progression as well as DNA damage checkpoints [42]. PLK1 depletion leads to varying lengths of mitotic delay and DNA damage [28, 29]. A previous *in vitro* study showed that inhibition or knock down of PLK1 in six cancer cell lines led to increased histone H3 phosphorylation (pHisH3) at 6 h followed by increased γ-H2AX expression at 24 h and increased PARP cleavage at 24–48 h, with senescence occurring in some of these cell lines after 2 weeks [29]. The timing of these changes suggests a model in which mitotic arrest leads to DNA damage and apoptosis and/or senescence. Yim, et. al.'s study showed that PLK1 knock down in HeLa cells led to DNA damage at the G1/S phase with increased ATM/CHK2 activation and γ-H2AX expression. The proposed mechanism is that the loss of PLK1 activity allows Emi1 accumulation, APC inactivation, and geminin accumulation, which lead to chromatin disruption and subsequent DNA damage [28]. Our results indicated that the co-administration of volasertib and erlotinib led to a strikingly increase in the expression of γ-H2AX, which reflects double-strand DNA breaks and DNA checkpoint ATR/CHK1 kinase activation. These results suggest that DNA damage is potentiated the lethality of PLK1 inhibition plus EGFR inhibition. EGFR and PI3K/AKT have been shown to enhance DNA repair after radiotherapy-induced DNA damage [43, 44]. The results of our study suggest that the addition of EGFR inhibition enhances the effect of PLK1 inhibition by further inhibiting DNA repair.

One limitation of our study was that we had relatively few ER lines in each category. Another limitation of our study is that we did not use an animal model with an intact immune system. Thus, we did not incorporate immune escape as a potential resistance mechanism [45]. The lack of heterogeneity is another limitation of laboratory resistance models.

Our findings suggest that PLK1 inhibition is therapeutically useful in NSCLC that becomes resistant to EGFR inhibition by EMT or acquisition of a *T790M* mutation. The two ER NSCLC cell lines that underwent EMT were sensitive to volasertib as a single agent. In contrast, the addition of volasertib overcame erlotinib resistance in all models with the *T790M* mutation. PLK1 inhibition combined with EGFR inhibition induced apoptosis by enhancing DNA damage. This combination strategy could be readily applied in the clinic for the treatment of NSCLC with acquired resistance to EGFR TKIs. Together, these results uncover new functions of PLK1 inhibition in the treatment of NSCLC with the *T790M* mutation that has acquired resistance to EGFR TKIs.

## MATERIALS AND METHODS

### Reagents

Erlotinib and volasertib were obtained from Selleck Chemicals (Houston, TX) and dissolved in DMSO as 10 mmol/L stock solutions. The mouse monoclonal anti-PLK1 antibody was purchased from Invitrogen (New York, NY). Antibodies against PLK1, E-cadherin, vimentin, PARP, p-EGFR(Tyr1068), EGFR, p-ERK(Tyr202/204), ERK, p-AKT(Ser473), AKT, γ-H2AX, p-ATM(Ser1981), ATM, p-ATR(Ser428), ATR, p-CHK1(Ser345), CHK1, p-CHK2(Tyr68), and CHK2 were purchased from Cell Signaling Technology (Danvers, MA).

### Cell culture and development of ER clones

Three *EGFR*-mutant, erlotinib-sensitive human lung adenocarcinoma cell lines (PC9, HCC4006, and HCC827) were obtained from American Type Culture Collection (Manassas, VA) and cultured in RPMI-1640 medium supplemented with 10% fetal bovine serum in a humidified 5% CO_2_ atmosphere at 37°C. ER cells were developed by subjecting these cells to chronic, repeated exposure to stepwise-increased concentrations of erlotinib as described previously [46]. The established ER cell lines were maintained in erlotinib at a concentration of 1 μM.

### Cell viability assays

NSCLC cells were seeded at 800–1000 cells/well in a 384-well plate (3 wells/sample, technical replicate). Each assay was repeated at least once on a separate day (biological replicate). Cells were treated with erlotinib and/or volasertib at various concentrations. After 72 h of treatment, cell viability was estimated using the CellTiter-Glo luminescent assay (Promega, Madison, WI) as previously described [16]. For each cell line, six technical replicates were tested at each concentration, and at least two biological replicates were tested on different days. IC_50_ and IC_70_ values were estimated from the best-fit dose-response model selected by residual standard error using the R package drexplorer [47].

### Western blot analysis

Cells were lysed in cell lysis buffer, and the cell lysates' protein concentrations were measured with the BCA Protein Assay Kit (Pierce Biotechnology, Rockford, IL) as previously described [35]. Samples containing 50 μg of protein were separated with 4–20% sodium dodecyl sulfate–polyacrylamide gel electrophoresis and transferred to polyvinylidene difluoride membranes (Bio-Rad Laboratories, Hercules, CA). The membranes were blocked in 5% milk/Tris-buffered saline plus Tween 20 for 1 h and then incubated with primary antibodies overnight at 4°C. The membranes were washed three times and incubated with specific secondary antibodies for 1 h. Bands were visualized using an enhanced chemiluminescent substrate (Pierce Biotechnology). Densitometry quantification of the bands was performed with the ImageJ software program (Version 1.48, National Institutes of Health, USA).

### Cell cycle and apoptosis analysis

NSCLC cell lines were treated with 60 nM volasertib and/or 2 μM erlotinib for 48 h for cell cycle analysis or 72 h for apoptosis analysis. For the cell cycle analysis, a BrdU FITC flow cytometry kit with 7-amino-actinomycin D (7AAD; BD Biosciences, San Jose, CA) was used according to the manufacturer's instructions. For the apoptosis analysis, the cells and the supernatant were collected, and an APO-BrdU TUNEL Assay kit (BD Biosciences) was used. Both the cell cycle analysis and the apoptosis analysis were performed as we described previously [36].

### Comet assay

DNA fragmentation was detected with a comet assay (Cell Biolabs, Inc., San Diego, CA) according to the manufacturer's instructions. PC9-ER9 cells at a concentration of 10^5^ cells/mL were mixed with 1% low temperature melting agarose at a ratio of 1:9 (v/v). Of this suspension, 75 μL was spread on a pre-coated slide and allowed to gel for 15 min at 4°C. The slide was then gently placed in precooled lysis solution (2.5 M NaCl, 100 mM EDTA, pH 10, 10 mM Tris base, 10% DMSO, 1% Triton X-100) for 30 min at 4°C. After lysis, the slides were equilibrated in alkaline solution (300 mM NaOH, pH > 13, 1 mM EDTA), and electrophoresis for 25 min at 30V was performed. Then, the slides were stained with Vista Green DNA Dye solution for 15 min and analyzed with fluorescence microscopy. For evaluation of the comet patterns, ~ 100 nuclei from each slide were analyzed by Comet Score Pro (TriTek Corp., Sumerduck, VA) and tail moment were calculated.

### Subcutaneous xenograft models

All animal research was conducted in accordance with The University of Texas MD Anderson Cancer Center's Institutional Animal Care and Use Committee. PC9-ER9 cells (2 × 10^6^ cells per mouse) were injected subcutaneously into the flanks of female nude mice (Harlan Laboratories, Indianapolis, IN). Tumors were measured by caliper twice weekly. Tumor volumes in millimeters were calculated with the formula (length × width^2^)/2. When tumor volumes reached 150 mm^3^, the mice were treated with intravenous injections of volasertib (30 mg/kg/week) and/or oral gavage of erlotinib (30 mg/kg/day). The control mice were treated with oral gavage of vehicle control daily and injection of vehicle control weekly. Mice were humanely killed after 3 weeks of treatment. Pairwise analysis was used to assess differences among the groups' treatment responses.

### Immunohistochemistry

Tissue specimens were fixed in formalin-buffer and embedded in paraffin (FFPE). From each FFPE sample, 4 microns sections were cut in a microtome and mounted on charged glass slides. One section was stained with hematoxylin-eosin (H&E) for pathology evaluation, and consecutive sections were stained for IHC with the following rabbit monoclonal antibodies: Ki67 (Cell Signaling Technology, clone D2H10, cat. # 9027, dilution 1:400); Cleaved Caspase-3 (Cell Signaling Technology, clone D3E9, cat. #9579, dilution 1:100). All IHC reactions were performed in a Leica Bond Max autostainer system (Leica Biosystems). Antigen retrieval was performed with BOND Epitope Retrieval Solution 1 (equivalent to citrate pH6.0, Leica Biosystems). Bond Polymer Refine Detection kit (Leica Biosystems) was employed as a detection system using a modified protocol removing the post-primary antibody step for the IHC staining of mouse xenografts in order to avoid background from mouse tissue IgG. Diaminobenzidine (DAB) was used as chromogen for the visualization of the IHC staining. The slides are then counterstained with hematoxylin, dehydrated and coverslipped. All IHC were performed with the respective positive controls (human tonsil for Ki67 and cleaved caspase-3). Additional control slides with positive and negative cell pellets were employed for cleaved caspase-3 (Cell Signaling Technology, cat. #8104). All IHC slides were evaluated for quality control by two pathologists (PV and JRC).

### Digital pathology analysis of the IHC slides

After IHC quality control, all the slides including H&E, IHC and control slides were scanned in an Aperio AT2 scanner (Leica Biosystems). IHC scoring was performed by a pathologist (PV) using Aperio Brightfield Toolbox image analysis software (Leica Biosystems). A nuclear algorithm were applied for the analysis of Ki67 and cleaved caspase-3. The data were presented as percentage of tumor cell nuclei positive. The final data, images and report were reviewed by two pathologists (PV and JR).

### Statistical analysis

The synergistic action of erlotinib and volasertib was quantified by the Chou-Talalay method using Calcusyn software (Biosoft, Cambridge, MA) [48]. The combination index (CI) of the two drugs, which was based on the growth inhibition effect of each drug alone or that of their combination was used to determine whether one drug had synergism (CI < 1), an additive effect (CI = 1), or antagonism (CI > 1) with the other.

IC_50_, IC_70_, and scaled AUC (area under curve) values were estimated using the best-fit dose-response model selected by the residual standard error using drexplorer [48]. AUC (0 for extreme sensitivity, 1 for extreme resistance) served as a measure of sensitivity reflecting both potency and total level of inhibition. One-way analysis of variance (ANOVA) was used to compare sensitivity between different groups. Spearman rank correlation was used to assess association between sensitivity and continuous variables.

The animal data included tumor size measurements at different times. We considered the correlations between the measurements on the same mouse. We fit this linear model (with treatment, time effect, and its interaction effect) using the generalized least squares method. The parameters were estimated by the maximizing restricted log-likelihood (REML) method. We used the Akaike information criterion (AIC) for selecting the best correlation structure. The analysis was performed using *nlme* package in R (version 3.1.3). Tukey's HSD (honest significant difference) test is used for post-hoc pairwise comparison. The analysis was performed using *nlme* and *multcomp* packages in R (version 3.1.3, https://www.r-project.org/).

For comet assay and IHC staining data one-way ANOVA is used for comparison in treatment groups. And post-hoc Tukey's HSD test is used for pairwise comparison.

## SUPPLEMENTARY MATERIALS AND FIGURES


