# Supplementary material for: Polo-like kinase 1 inhibition diminishes acquired resistance to epidermal growth factor receptor inhibition in non-small cell lung cancer with T790M mutations
Source: Oncotarget. 2016 Jun 30;7(30):47998–8010. doi: 10.18632/oncotarget.10332 (PMC5216995; doi:10.18632/oncotarget.10332)
Supplement: Supplementary file 1 [file oncotarget-07-47998-s001.pdf]

Polo-like kinase 1 inhibition diminishes acquired resistance to epidermal growth factor receptor inhibition in non-small cell lung cancer with *T790M* mutations

Supplementary Materials

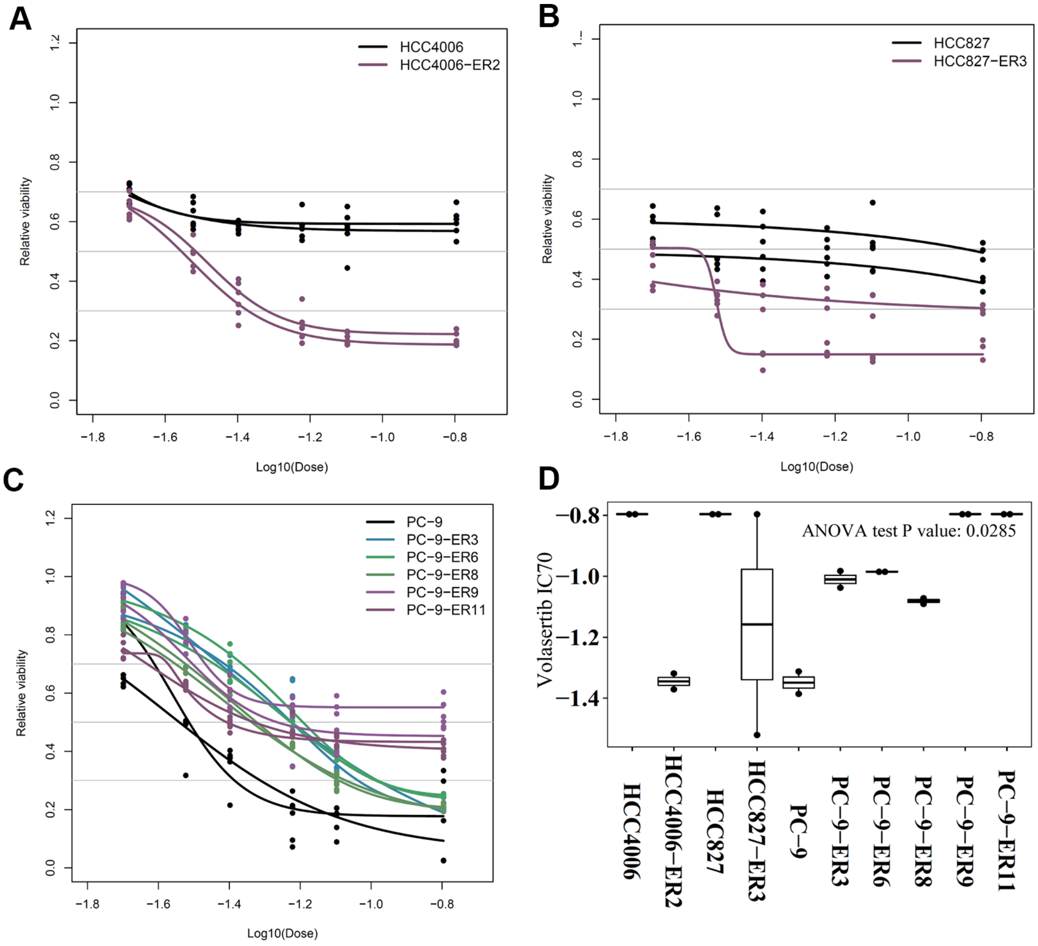

Supplementary Figure S1: Volasertib dose-response curves of parental and ER NSCLC cell lines. Parental and ER NSCLC cell lines were incubated with the indicated concentrations of volasertib, and cells were quantitated with the CellTiter-Glo assay. Each assay was completed on two different days with three technical replicates.

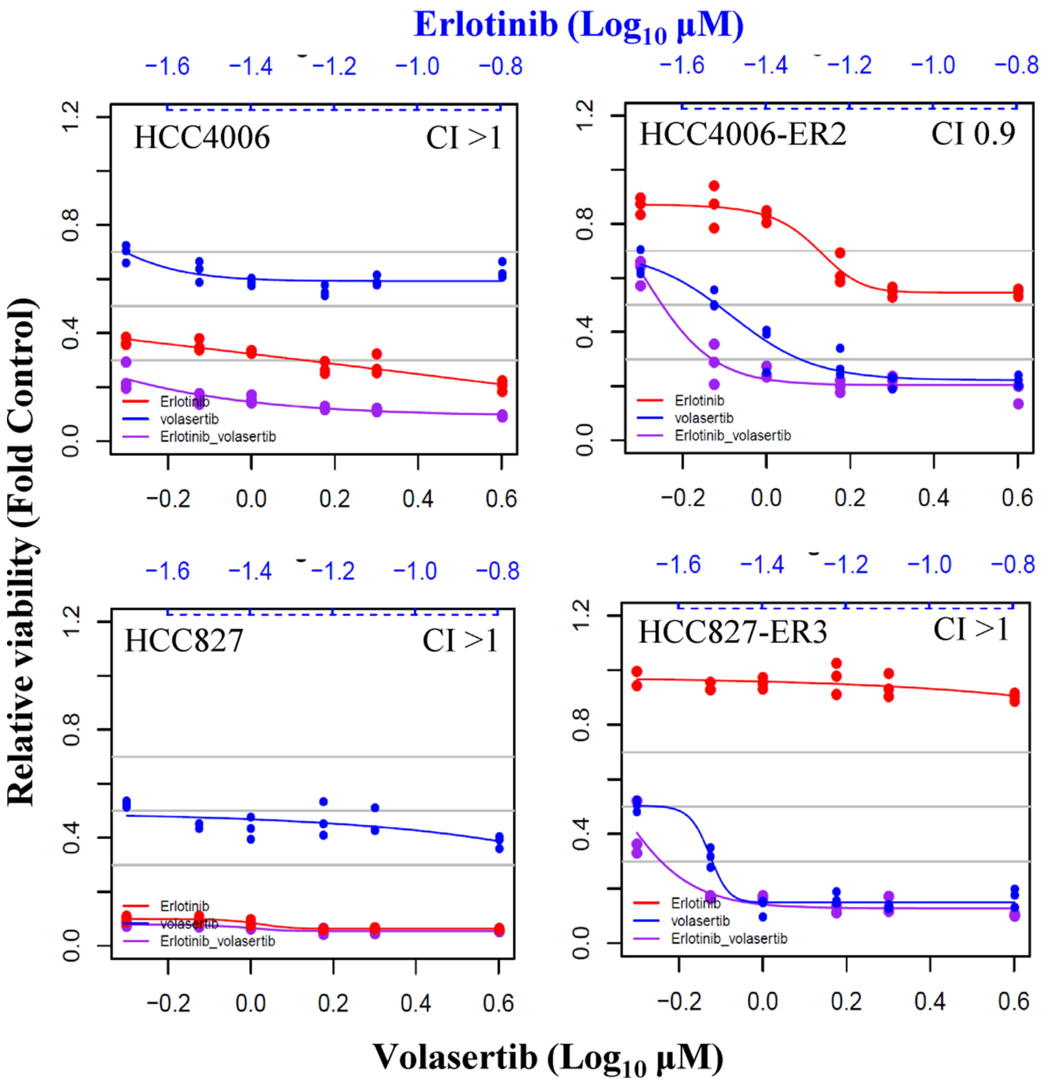

Supplementary Figure S2: PLK1 inhibition plus EGFR inhibition in ER HCC4006 and HCC827 cell lines. The viability of ER cell lines treated with the indicated doses of volasertib and/or erlotinib for 72 h was determined with a CellTiter-Glo assay. The CI of the two drugs was calculated using the Calcusyn software program. CI depicts synergism (CI < 1), additive effect (CI = 1), and antagonism (CI > 1).
